# Crystal structure and Hirshfeld surface analysis of (*E*)-1-[2,2-di­chloro-1-(4-fluoro­phen­yl)ethen­yl]-2-(2,4-di­chloro­phen­yl)diazene

**DOI:** 10.1107/S2056989021010756

**Published:** 2021-10-26

**Authors:** Namiq Q. Shikhaliyev, Kadiriye Özkaraca, Mehmet Akkurt, Xanim N. Bagirova, Gulnar T. Suleymanova, Mirjalil S. Abdulov, Sixberth Mlowe

**Affiliations:** a Organic Chemistry Department, Baku State University, Z. Khalilov str. 23, AZ 1148 Baku, Azerbaijan; b Institute of Natural and Applied Science, Erciyes University, 38039 Kayseri, Turkey; c Department of Physics, Faculty of Sciences, Erciyes University, 38039 Kayseri, Turkey; d University of Dar es Salaam, Dar es Salaam University College of Education, Department of Chemistry, PO Box 2329, Dar es Salaam, Tanzania

**Keywords:** crystal structure, short inter H*L*⋯H*L* contact, C—Cl⋯π inter­actions, face-to-face π–π stacking inter­actions, Hirshfeld surface analysis

## Abstract

In the title compound, C_14_H_7_Cl_4_FN_2_, the dihedral angle between the 4-fluoro­phenyl ring and the 2,4-di­chloro­phenyl ring is 46.03 (19)°. In the crystal, the mol­ecules are linked by C—H⋯N inter­actions along the *a*-axis direction, forming a *C*(6) chain. The mol­ecules are further connected by C—Cl⋯π inter­actions and face-to-face π–π stacking inter­actions, forming ribbons along the *a*-axis direction. Hirshfeld surface analysis indicates that the greatest contributions to the crystal packing are from Cl⋯H/H⋯Cl (35.1%), H⋯H (10.6%), C⋯C (9.7%), Cl⋯Cl (9.4%) and C⋯H/H⋯C (9.2%) inter­actions.

## Abstract 2

In the crystal, C—H⋯N, C—Cl⋯π inter­actions and face-to-face π–π stacking inter­actions connect the mol­ecules, forming ribbons along the *a-*axis direction.

## Chemical context

Azo dyes find numerous applications in a diversity of areas, including as anti­microbial agents, in mol­ecular recognition, optical data storage, mol­ecular switches, non-linear optics, liquid crystals, dye-sensitized solar cells, color-changing materials, *etc*., mainly due to the possibility of the *cis-*to-*trans* isomerization and the chromophoric properties of the –N=N– synthon (Maharramov *et al.*, 2018 ▸; Viswanathan *et al.*, 2019 ▸). Not only azo-hydrazone tautomerisim, but also *E/Z* isomerization are important phenomena in the synthetic chemistry of azo dyes (Ma *et al.*, 2017*a* ▸,*b* ▸; Mahmoudi *et al.*, 2018*a* ▸,*b* ▸). The design of azo dyes with functional groups led to multifunctional ligands, the corresponding transition-metal complexes of which have been used effectively as catalysts in C—C coupling and oxidation reactions (Ma *et al.*, 2020 ▸, 2021 ▸; Mahmudov *et al.*, 2013 ▸; Mizar *et al.*, 2012 ▸). Moreover, the functional properties of azo dyes can be improved by attaching substituents with non-covalent bond donor or acceptor site(s) to the –N=N– synthon (Gurbanov *et al.*, 2020*a* ▸,*b* ▸; Kopylovich *et al.*, 2011 ▸; Mahmudov *et al.*, 2020 ▸; Shixaliyev *et al.*, 2014 ▸). Thus, we have attached halogen-bond donor centres to the –N=N– moiety, leading to a new azo dye, (*E*)-1-[2,2-di­chloro-1-(4-fluoro­phen­yl)ethen­yl]-2-(2,4-di­chloro­phen­yl)diazene, which provides multiple inter­molecular non-covalent inter­actions.

## Structural commentary

**Figure 1:**
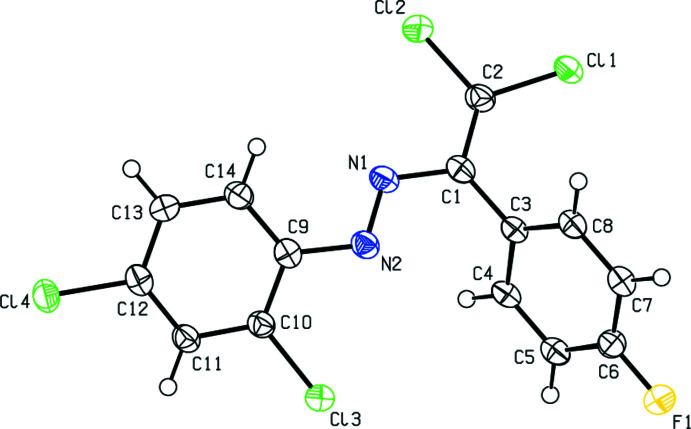
The mol­ecular structure of the title compound, showing the atom labelling and displacement ellipsoids drawn at the 50% probability level.

In the title compound, (Fig. 1 ▸), the dihedral angle between the 4-fluoro­phenyl ring C3–C8 and the 2,4-di­chloro­phenyl ring C9–C14 is 46.0 (2)°. The N2/N1/C1/C2/Cl1/Cl2 moiety is approximately planar, with a maximum deviation of 0.029 (1) Å for Cl1, and makes dihedral angles of 50.53 (18) and 11.75 (18)° with the C3–C8 and C9–C14 rings, respectively. In the mol­ecule, the aromatic ring and olefin synthon adopt a *trans*-configuration with respect to the N=N double bond and are almost coplanar with a C1—N1=N2—C9 torsion angle of 179.1 (4)°. 
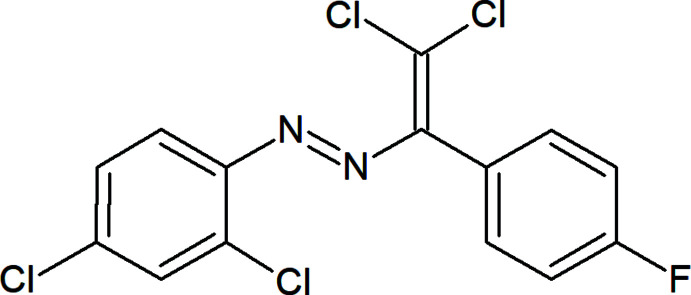


## Supra­molecular features

**Figure 2:**
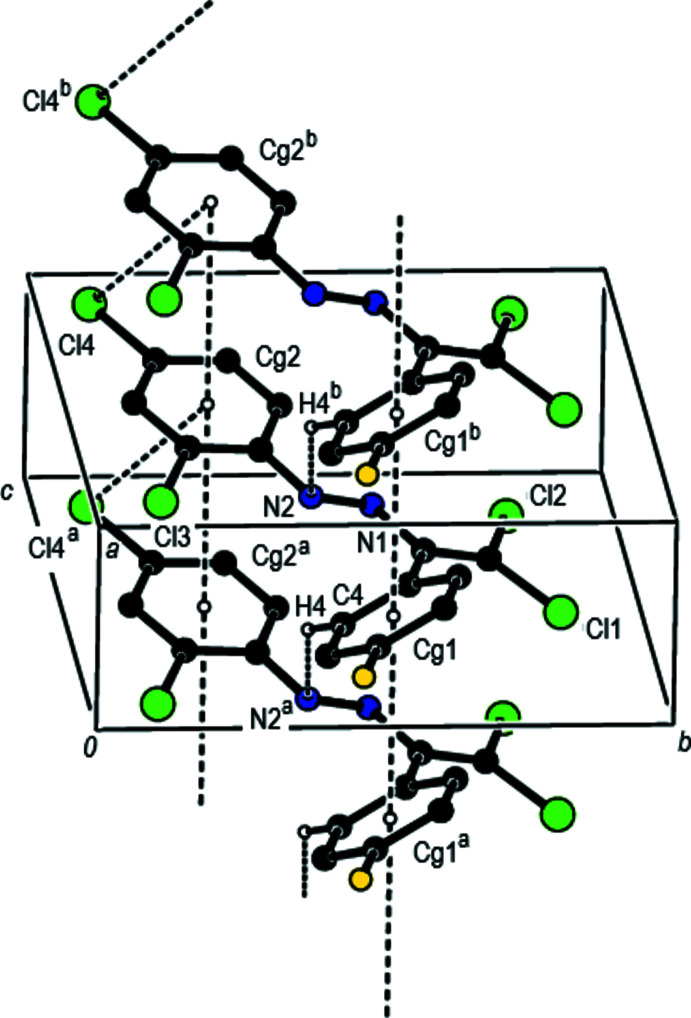
A general view of the inter­molecular C—H⋯N and C—Cl⋯π inter­actions and π–π stacking inter­actions, shown as dashed lines. Symmetry codes: (*a*) − 1 + *x*, *y*, *z*; (*b*) 1 + *x*, *y*, *z*.

In the crystal, the mol­ecules are linked by C—H⋯N inter­actions along the *a*-axis direction, forming a *C*(6) chain (Table 1 ▸; Fig. 2 ▸; Bernstein *et al.*, 1995 ▸). Furthermore, mol­ecules are connected by C—Cl⋯*Cg*2 inter­actions (Table 1 ▸) and face-to-face π-π stacking inter­actions [*Cg*1⋯*Cg*1^i^ = 3.873 (3) Å, slippage = 1.831 Å; *Cg*2⋯*Cg*2^i^ = 3.872 (3) Å, slippage = 1.554 Å; symmetry codes: (i) *x* − 1, *y*, *z*;; (ii) *x* + 1, *y*, *z*; where *Cg*1 and *Cg*2 are the centroids of the 4-fluoro­phenyl (C3–C8) and 2,4-di­chloro­phenyl ring (C9–C14) rings, respectively], forming ribbons along the *a*-axis direction (Figs. 2 ▸, 3 ▸ and 4 ▸).

## Hirshfeld surface analysis

**Figure 5:**
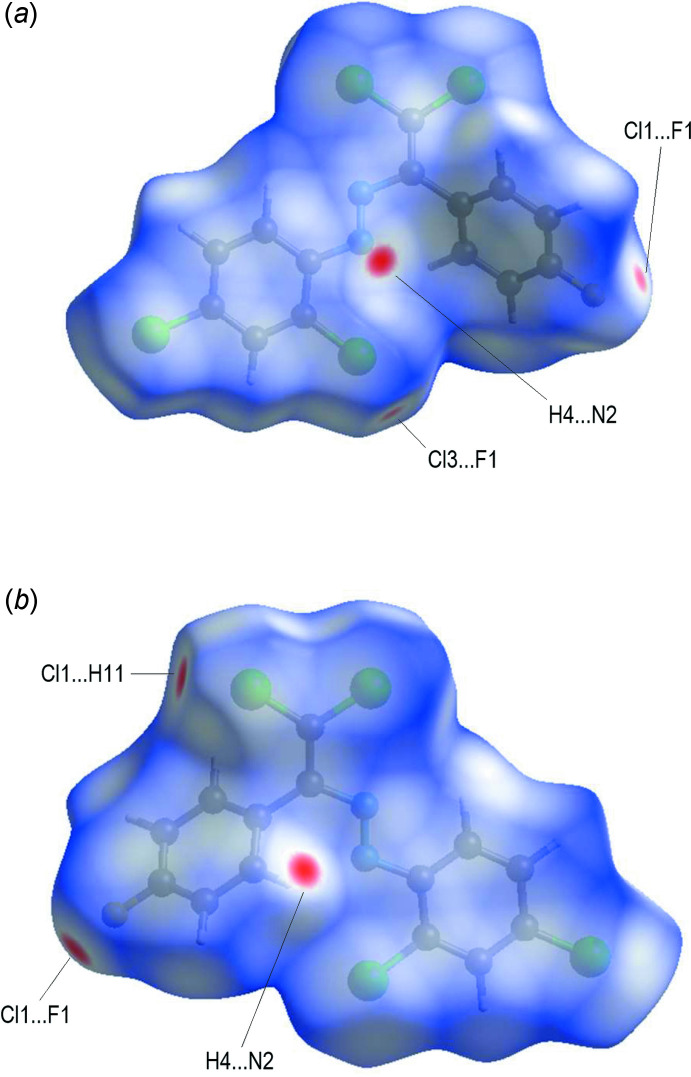
(*a*) Front and (*b*) back sides of the three-dimensional Hirshfeld surface of the title compound plotted over *d*_norm_ in the range −0.1450 to 1.1580 a.u.

**Figure 6:**
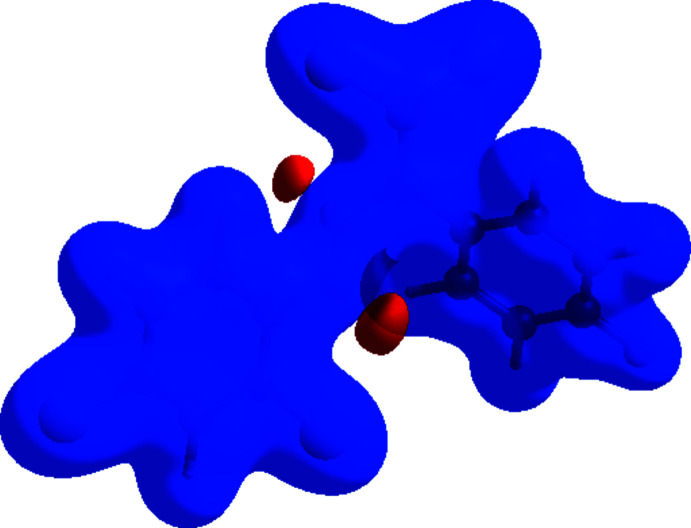
View of the three-dimensional Hirshfeld surface of the title compound plotted over electrostatic potential energy in the range −0.0500 to 0.0500 a.u. using the *STO-3 G* basis set at the Hartree–Fock level of theory. Hydrogen-bond donors and acceptors are shown as blue and red regions around the atoms, corresponding to positive and negative potentials, respectively.

*Crystal Explorer* (Turner *et al.*, 2017 ▸) was used to perform a Hirshfeld surface analysis and generate the associated two-dimensional fingerprint plots, with a standard resolution of the three-dimensional *d*_norm_ surfaces plotted over a fixed colour scale of −0.1450 (red) to 1.1580 (blue) a.u (Fig. 5 ▸). In the Hirshfeld surface mapped over *d*_norm_ (Fig. 5 ▸), the bright-red spots near atoms Cl1, Cl3, H4, N2 and F1 indicate the short C—H⋯N, C—H⋯Cl and Cl⋯F contacts (Table 2 ▸). Other contacts are equal to or longer than the sum of van der Waals radii. The Hirshfeld surface of the title compound mapped over the electrostatic potential (Spackman *et al.*, 2008 ▸) is shown in Fig. 6 ▸. The positive electrostatic potential (blue regions) over the surface indicates hydrogen-donor potential, whereas the hydrogen-bond acceptors are represented by negative electrostatic potential (red regions).

**Figure 7:**
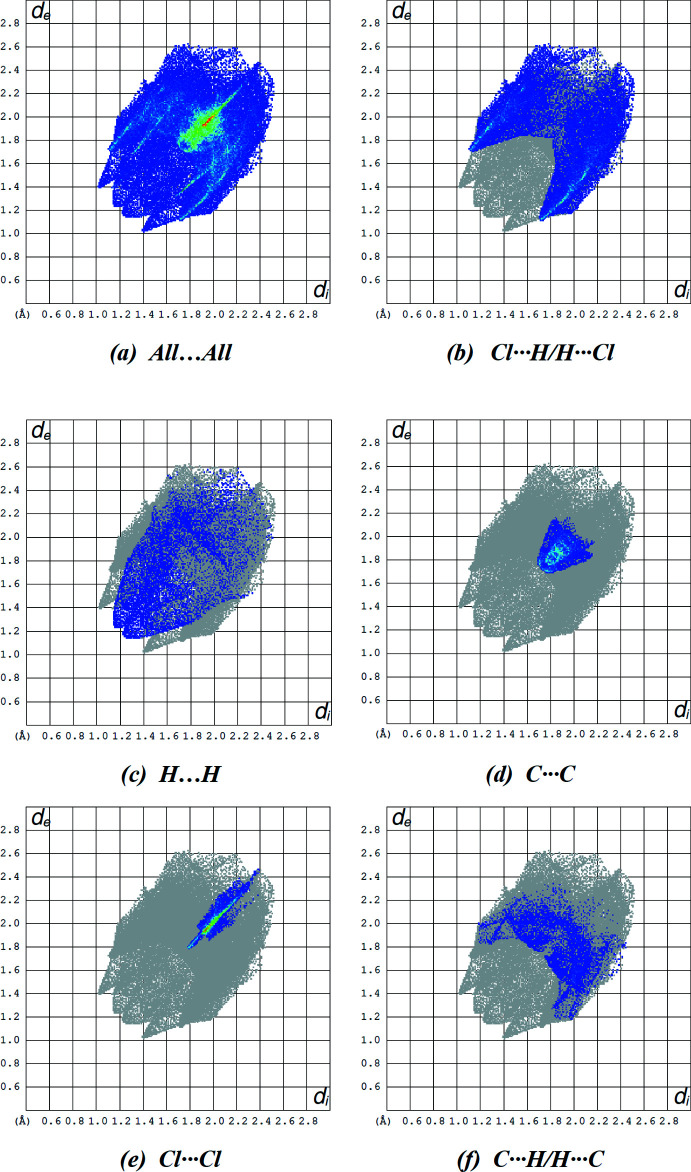
The full two-dimensional fingerprint plot for the title compound and those delineated into (*b*) Cl⋯H/H⋯Cl (35.1%), (*c*) H⋯H (10.6%), (*d*) C⋯C (9.7%), (*e*) Cl⋯Cl (9.4%) and (*f*) C⋯H/H⋯C (9.2%) inter­actions.

The overall two-dimensional fingerprint plot and those delineated into Cl⋯H/H⋯Cl, H⋯H, C⋯C, Cl⋯Cl and C⋯H/H⋯C contacts in the title mol­ecule are illustrated in Fig. 7 ▸. The most important inter­action is Cl⋯H/H⋯Cl, contributing 35.1% to the overall crystal packing (Fig. 7 ▸ *b*). The secondary important H⋯H and C⋯C inter­actions contribute 10.6% (Fig. 7 ▸ *c*) and 9.7% (Fig. 7 ▸ *d*), respectively, to the Hirshfeld surface. The remaining contributions for the title compound are from Cl⋯Cl, C⋯H/H⋯C, Cl⋯F/F⋯Cl, Cl⋯C/C⋯Cl, F⋯H/H⋯F, N⋯H/H⋯N, N⋯N and F⋯C/ C⋯F contacts, which are less than 9.7% and have a negligible effect on the packing. The percentage contributions of all inter­actions are listed in Table 3 ▸.

## Database survey

A search of the Cambridge Structural Database (CSD, Version 5.41, update of November 2019; Groom *et al.*, 2016 ▸) for the (*E*)-1-(2,2-di­chloro-1-phenyl­ethen­yl)-2-phenyl­diazene unit resulted in 28 hits. Nine compounds are closely related to the title compound, *viz*. LEQXOX (**I**; Shikhaliyev *et al.*, 2018 ▸), LEQXIR (**II**; Shikhaliyev *et al.*, 2018 ▸), XIZREG (**III**; Atioğlu *et al.*, 2019 ▸), HODQAV (**IV**; Shikhaliyev *et al.*, 2019 ▸), HONBUK (**V**; Akkurt *et al.*, 2019 ▸), HONBOE (**VI**; Akkurt *et al.*, 2019 ▸), DULTAI (**VII**; Özkaraca *et al.*, 2020*b* ▸), GUPHIL (**VIII**; Özkaraca *et al.*, 2020*a* ▸) and EBUCUD (**IX**; Shikhaliyev *et al.*, 2021 ▸).

In the crystals of **I** and **II**, the dihedral angles between the aromatic rings are 56.18 (12) and 60.31 (14)°, respectively. In **I**, C—H⋯N and short Cl⋯Cl contacts are observed and in **II**, C—H⋯N and C—H⋯O hydrogen bonds and short C—Cl⋯O contacts occur. In **III**, the benzene rings form a dihedral angle of 63.29 (8)° and the mol­ecules are linked by C—H⋯O hydrogen bonds into zigzag chains running along the *c*-axis direction. The crystal packing also features C—Cl⋯π, C—F⋯π and N—O⋯π inter­actions. In **IV**, the benzene rings make a dihedral angle of 56.13 (13)°. Mol­ecules are stacked in columns along the *a*-axis direction *via* weak C—H⋯Cl hydrogen bonds and face-to-face π–π stacking inter­actions. The crystal packing is further consolidated by short Cl⋯Cl contacts. In **V** and **VI**, the aromatic rings form dihedral angles of 60.9 (2) and 64.1 (2)°, respectively. In the crystals, mol­ecules are linked through weak *X*⋯Cl contacts (*X* = Cl for **V** and Br for **VI**), C—H⋯Cl and C—Cl⋯π inter­actions into sheets parallel to the *ab* plane. Additional van der Waals inter­actions consolidate the three-dimensional packing. In **VII**, the dihedral angle between the two aromatic rings is 64.12 (14)°. The crystal structure is stabilized by a short C—H⋯Cl contact, C—Cl⋯π and van der Waals inter­actions. In **VIII**, the benzene rings subtend a dihedral angle of 77.07 (10)°. In the crystal, mol­ecules are associated into inversion dimers *via* short Cl⋯Cl contacts [3.3763 (9) Å]. In **IX**, the asymmetric unit comprises two similar mol­ecules, in which the dihedral angles between the two aromatic rings are 70.1 (3) and 73.2 (2)°. The crystal structure features short C—H⋯Cl and C—H⋯O contacts and C—H⋯π and van der Waals inter­actions.

## Synthesis and crystallization

The title dye was synthesized according to the reported method (Shikhaliyev *et al.*, 2018 ▸, 2019 ▸). A 20 mL screw-neck vial was charged with DMSO (10 mL), (*E*)-1-(2,4-di­chloro­phen­yl)-2-(4-fluoro­benzyl­idene)hydrazine (283 mg, 1 mmol), tetra­methyl­ethylenedi­amine (TMEDA) (295 mg, 2.5 mmol), CuCl (2 mg, 0.02 mmol) and CCl_4_ (20 mmol, 10 equiv.). After 1–3 h (until TLC analysis showed complete consumption of the corresponding Schiff base), the reaction mixture was poured into ∼0.01 *M* solution of HCl (100 mL, pH = 2–3), and extracted with di­chloro­methane (3 × 20 mL). The combined organic phase was washed with water (3 × 50 mL) and brine (30 mL), dried over anhydrous Na_2_SO_4_ and concentrated using a vacuum rotary evaporator. The residue was purified by column chromatography on silica gel using appropriate mixtures of hexane and di­chloro­methane (3/1–1/1). Crystals suitable for X-ray analysis were obtained by slow evaporation of an ethanol solution. Colourless solid (44%); m.p. 345 K. Analysis calculated for C_14_H_7_Cl_4_FN_2_ (*M* = 364.02): C 46.19, H 1.94, N 7.70; found: C 46.11, H 1.98, N 7.67%. ^1^H NMR (300 MHz, CDCl_3_) *δ* 7.31–7.83 (7H, Ar). ^13^C NMR (75 MHz, CDCl_3_) δ 114.89, 115.12, 115.41, 115.74, 115.97, 118.33, 127.73, 128.08, 128.67, 129.17, 130.48, 132.04, 132.15 and 136.83. ESI–MS: *m*/*z*: 365.11 [*M* + H]^+^.

## Refinement details

Crystal data, data collection and structure refinement details are summarized in Table 4 ▸. The Moscow synchrotron radiation source was used for the data collection. H atoms were positioned geometrically and treated as riding atoms where C—H = 0.95 Å with *U*_iso_(H) = 1.2*U*_eq_(C). Five outliers 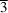 2 2, 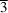 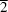 2, 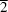 11 3, 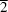 2 1 and 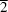 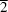 1 were omitted during the final refinement cycle because of large differences between observed and calculated intensities.

## Supplementary Material

Crystal structure: contains datablock(s) I. DOI: 10.1107/S2056989021010756/vm2255sup1.cif

Structure factors: contains datablock(s) I. DOI: 10.1107/S2056989021010756/vm2255Isup2.hkl

Supporting information file. DOI: 10.1107/S2056989021010756/vm2255Isup3.cml

CCDC reference: 2116300

Additional supporting information:  crystallographic information; 3D view; checkCIF report

## Figures and Tables

**Figure 3:**
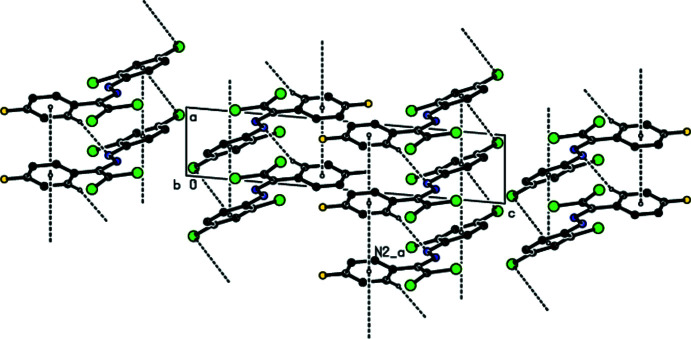
The crystal packing of the title compound viewed along the *b* axis with inter­molecular C—H⋯N and C—Cl⋯π inter­actions and π–π stacking inter­actions shown as dashed lines.

**Figure 4:**
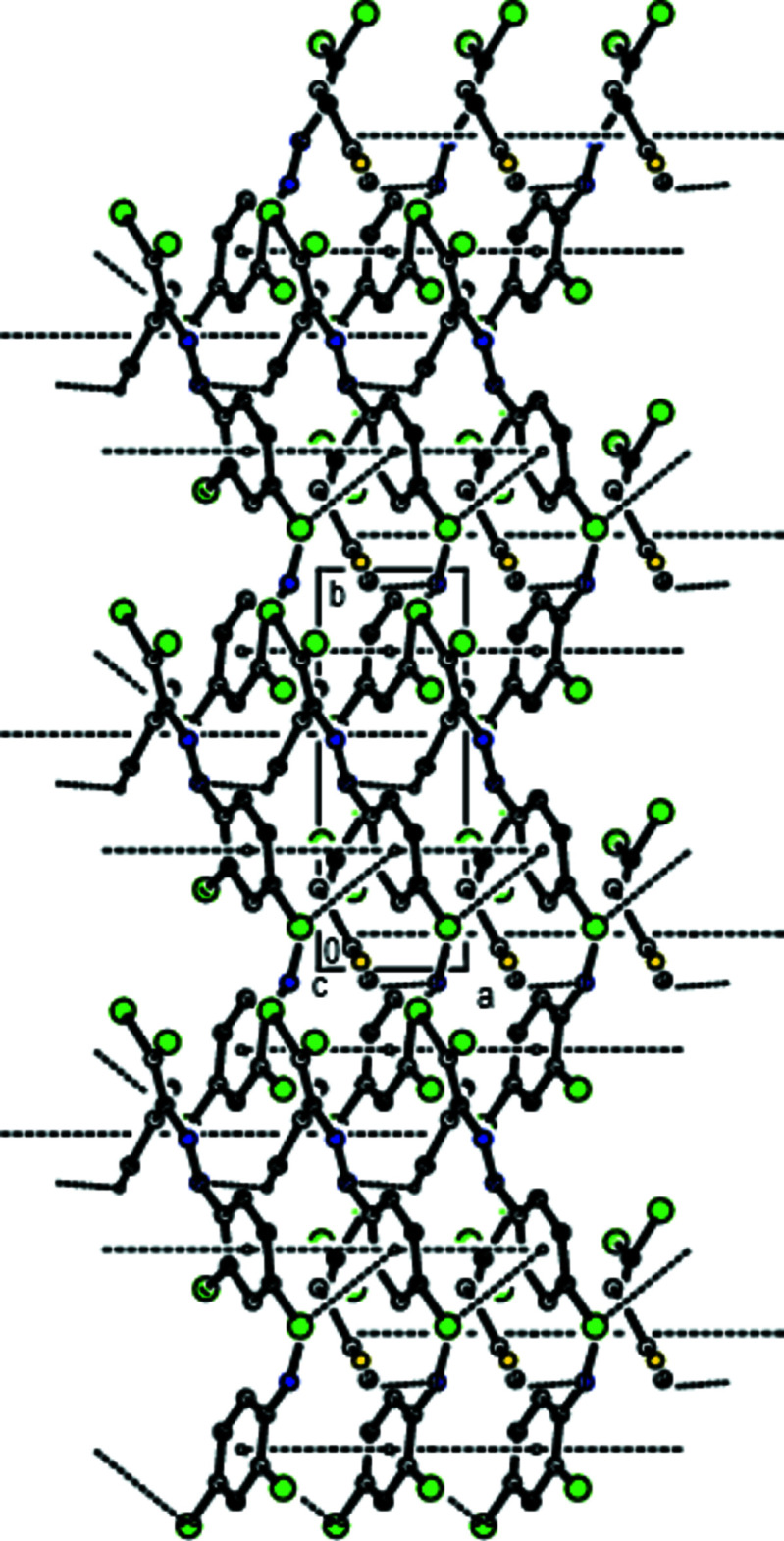
The crystal packing of the title compound viewed along the *c* axis with inter­molecular C—H⋯N and C—Cl⋯π inter­actions and π-π stacking inter­actions shown as dashed lines.

**Table 1:** Hydrogen-bond geometry (Å, °) *Cg*2 is the centroid of the 2,4-di­chloro­phenyl ring (C9–C14).

*D*—H⋯*A*	*D*—H	H⋯*A*	*D*⋯*A*	*D*—H⋯*A*
C4—H4⋯N2^i^	0.95	2.53	3.265 (5)	134
C12—Cl4⋯*Cg*2^ii^	1.735 (5)	3.920 (3)	3.569 (6)	66.51 (18)

Symmetry codes: (i) x-1, y, z; (ii) x+1, y, z.

**Table 2:** Summary of short inter­atomic contacts (Å) in the title compound

Contact	Distance	Symmetry operation
Cl1⋯H11	3.06	−1 + *x*, 1 + *y*, *z*
H4⋯N2	2.53	−1 + *x*, *y*, *z*
Cl1⋯F1	3.016 (3)	−1 − *x*, {1\over 2} + *y*, 1 − *z*
H5⋯H7	2.55	−*x*, −{1\over 2} + *y*, 1 − *z*
Cl4⋯H13	2.95	2 − *x*, −{1\over 2} + *y*, 2 − *z*
Cl4⋯H14	2.93	1 − *x*, −{1\over 2} + *y*, 2 − *z*
Cl3⋯F1	3.116 (3)	−*x*, −{1\over 2} + *y*, 1 − *z*

**Table 3:** Percentage contributions of inter­atomic contacts to the Hirshfeld surface for the title compound.

Contact	Percentage contribution
Cl⋯H/H⋯Cl	35.1
H⋯H	10.6
C⋯C	9.7
Cl⋯Cl	9.4
C⋯H/H⋯C	9.2
Cl⋯F/F⋯Cl	6.7
Cl⋯C/C⋯Cl	5.0
F⋯H/H⋯F	5.0
N⋯H/H⋯N	4.4
N⋯C/C⋯N	3.5
F⋯F	0.9
N⋯N	0.3
F⋯C/C⋯F	0.1

**Table 4:** Experimental details

Crystal data	
Chemical formula	C_14_H_7_Cl_4_FN_2_
*M*_r_	364.02
Crystal system, space group	Monoclinic, *P*2_1_
Temperature (K)	100
*a*, *b*, *c* (Å)	3.8720 (8), 10.434 (2), 18.138 (4)
β (°)	95.03 (3)
*V* (Å^3^)	730.0 (3)
*Z*	2
Radiation type	Synchrotron, λ = 0.79475 Å
μ (mm^−1^)	1.10
Crystal size (mm)	0.20 × 0.15 × 0.10

Data collection	
Diffractometer	Rayonix SX165 CCD
Absorption correction	Multi-scan (*SCALA*; Evans, 2006 ▸)
*T*_min_, *T*_max_	0.800, 0.880
No. of measured, independent and observed [*I* > 2σ(*I*)] reflections	8595, 3120, 2972
*R*_int_	0.027
(sin θ/λ)_max_ (Å^−1^)	0.648

Refinement	
*R*[*F* ^2^ > 2σ(*F* ^2^)], *wR*(*F* ^2^), *S*	0.036, 0.106, 1.09
No. of reflections	3120
No. of parameters	191
No. of restraints	1
H-atom treatment	H-atom parameters constrained
Δρ_max_, Δρ_min_ (e Å^−3^)	0.61, −0.30
Absolute structure	Flack *x* determined using 1318 quotients [(*I* ^+^)−(*I* ^−^)]/[(*I* ^+^)+(*I* ^−^)] (Parsons *et al.*, 2013 ▸)
Absolute structure parameter	0.04 (2)

Computer programs: *Marccd* (Doyle, 2011 ▸), *iMosflm* (Battye *et al.*, 2011 ▸), *SHELXT* (Sheldrick, 2015*a* ▸), *SHELXL* (Sheldrick, 2015*b* ▸), *ORTEP-3 for Windows* (Farrugia, 2012 ▸) and *PLATON* (Spek, 2020 ▸).

## supplementary crystallographic information

### Crystal data

**Table:**

C_14_H_7_Cl_4_FN_2_	*F*(000) = 364
*M**_r_* = 364.02	*D*_x_ = 1.656 Mg m^−^^3^
Monoclinic, *P*2_1_	Synchrotron radiation, λ = 0.79475 Å
*a* = 3.8720 (8) Å	Cell parameters from 600 reflections
*b* = 10.434 (2) Å	θ = 2.8–28.0°
*c* = 18.138 (4) Å	µ = 1.12 mm^−^^1^
β = 95.03 (3)°	*T* = 100 K
*V* = 730.0 (3) Å^3^	Prism, colourless
*Z* = 2	0.20 × 0.15 × 0.10 mm

### Data collection

**Table:**

Rayonix SX165 CCD diffractometer	2972 reflections with *I* > 2σ(*I*)
/f scan	*R*_int_ = 0.027
Absorption correction: multi-scan (Scala; Evans, 2006)	θ_max_ = 31.0°, θ_min_ = 2.5°
*T*_min_ = 0.800, *T*_max_ = 0.880	*h* = −5→5
8595 measured reflections	*k* = −12→13
3120 independent reflections	*l* = −23→23

### Refinement

**Table:**

Refinement on *F*^2^	H-atom parameters constrained
Least-squares matrix: full	*w* = 1/[σ^2^(*F*_o_^2^) + (0.0549*P*)^2^ + 0.7552*P*] where *P* = (*F*_o_^2^ + 2*F*_c_^2^)/3
*R*[*F*^2^ > 2σ(*F*^2^)] = 0.036	(Δ/σ)_max_ < 0.001
*wR*(*F*^2^) = 0.106	Δρ_max_ = 0.61 e Å^−^^3^
*S* = 1.09	Δρ_min_ = −0.30 e Å^−^^3^
3120 reflections	Extinction correction: SHELXL, Fc^*^=kFc[1+0.001xFc^2^λ^3^/sin(2θ)]^-1/4^
191 parameters	Extinction coefficient: 0.044 (8)
1 restraint	Absolute structure: Flack *x* determined using 1318 quotients [(*I*^+^)-(*I*^-^)]/[(*I*^+^)+(*I*^-^)] (Parsons et al., 2013)
Hydrogen site location: inferred from neighbouring sites	Absolute structure parameter: 0.04 (3)

### Special details

**Table:**

Geometry. All esds (except the esd in the dihedral angle between two l.s. planes) are estimated using the full covariance matrix. The cell esds are taken into account individually in the estimation of esds in distances, angles and torsion angles; correlations between esds in cell parameters are only used when they are defined by crystal symmetry. An approximate (isotropic) treatment of cell esds is used for estimating esds involving l.s. planes.

### Fractional atomic coordinates and isotropic or equivalent isotropic displacement parameters (Å^2^)

**Table:**

	*x*	*y*	*z*	*U*_iso_*/*U*_eq_	
Cl1	−0.3214 (3)	0.89506 (12)	0.70389 (6)	0.0309 (3)	
Cl2	−0.0221 (4)	0.81658 (13)	0.84672 (7)	0.0392 (3)	
Cl3	0.2392 (3)	0.19949 (12)	0.70702 (6)	0.0334 (3)	
Cl4	0.8795 (3)	0.10530 (13)	0.97679 (6)	0.0340 (3)	
F1	−0.2885 (9)	0.5192 (3)	0.42861 (16)	0.0383 (7)	
N1	0.1181 (11)	0.5745 (4)	0.7820 (2)	0.0281 (9)	
N2	0.1962 (11)	0.4658 (4)	0.7569 (2)	0.0273 (8)	
C1	−0.0396 (12)	0.6583 (5)	0.7275 (3)	0.0274 (9)	
C2	−0.1180 (12)	0.7756 (5)	0.7555 (3)	0.0293 (10)	
C3	−0.1089 (12)	0.6232 (5)	0.6480 (2)	0.0255 (9)	
C4	−0.2763 (12)	0.5073 (5)	0.6280 (3)	0.0267 (9)	
H4	−0.347529	0.452035	0.665430	0.032*	
C5	−0.3391 (13)	0.4726 (5)	0.5540 (3)	0.0288 (10)	
H5	−0.455606	0.394787	0.540530	0.035*	
C6	−0.2292 (13)	0.5531 (5)	0.5008 (3)	0.0295 (10)	
C7	−0.0634 (12)	0.6678 (5)	0.5181 (3)	0.0289 (10)	
H7	0.006934	0.722093	0.480114	0.035*	
C8	−0.0016 (12)	0.7022 (5)	0.5920 (3)	0.0281 (9)	
H8	0.114818	0.780287	0.604727	0.034*	
C9	0.3594 (11)	0.3839 (5)	0.8125 (2)	0.0261 (9)	
C10	0.3960 (12)	0.2556 (5)	0.7935 (3)	0.0265 (9)	
C11	0.5577 (13)	0.1679 (5)	0.8440 (3)	0.0275 (9)	
H11	0.581566	0.080365	0.830976	0.033*	
C12	0.6813 (13)	0.2120 (5)	0.9131 (3)	0.0291 (10)	
C13	0.6495 (12)	0.3405 (5)	0.9334 (3)	0.0289 (10)	
H13	0.736480	0.368972	0.981195	0.035*	
C14	0.4893 (13)	0.4255 (5)	0.8827 (3)	0.0300 (10)	
H14	0.467401	0.513039	0.895888	0.036*	

### Atomic displacement parameters (Å^2^)

**Table:**

	*U*^11^	*U*^22^	*U*^33^	*U*^12^	*U*^13^	*U*^23^
Cl1	0.0349 (6)	0.0218 (6)	0.0353 (6)	0.0044 (5)	−0.0005 (4)	−0.0001 (4)
Cl2	0.0528 (8)	0.0325 (7)	0.0312 (6)	0.0116 (6)	−0.0029 (5)	−0.0065 (5)
Cl3	0.0426 (6)	0.0263 (6)	0.0301 (6)	0.0037 (5)	−0.0030 (4)	−0.0036 (4)
Cl4	0.0385 (6)	0.0315 (7)	0.0318 (5)	0.0058 (5)	0.0018 (4)	0.0071 (5)
F1	0.0507 (18)	0.0339 (18)	0.0296 (15)	0.0000 (14)	0.0002 (12)	−0.0029 (12)
N1	0.031 (2)	0.022 (2)	0.032 (2)	0.0034 (16)	0.0042 (15)	−0.0011 (15)
N2	0.028 (2)	0.025 (2)	0.0291 (19)	0.0041 (15)	0.0052 (15)	0.0005 (15)
C1	0.027 (2)	0.023 (2)	0.033 (2)	0.0038 (17)	0.0032 (17)	0.0004 (18)
C2	0.027 (2)	0.027 (3)	0.034 (2)	0.0060 (18)	0.0015 (18)	−0.0031 (19)
C3	0.027 (2)	0.020 (2)	0.029 (2)	0.0037 (17)	0.0026 (16)	0.0005 (17)
C4	0.028 (2)	0.019 (2)	0.034 (2)	0.0029 (17)	0.0062 (17)	0.0004 (17)
C5	0.029 (2)	0.022 (2)	0.036 (2)	0.0031 (17)	0.0024 (18)	0.0000 (18)
C6	0.030 (2)	0.030 (3)	0.028 (2)	0.0053 (18)	0.0009 (17)	−0.0018 (17)
C7	0.028 (2)	0.025 (3)	0.033 (2)	0.0024 (17)	0.0034 (17)	0.0035 (18)
C8	0.028 (2)	0.023 (2)	0.033 (2)	0.0029 (19)	0.0019 (16)	0.0018 (19)
C9	0.024 (2)	0.026 (2)	0.029 (2)	0.0012 (18)	0.0052 (15)	0.0031 (18)
C10	0.029 (2)	0.024 (2)	0.027 (2)	0.0040 (18)	0.0044 (17)	−0.0001 (17)
C11	0.029 (2)	0.024 (3)	0.030 (2)	0.0045 (17)	0.0046 (16)	0.0013 (17)
C12	0.029 (2)	0.027 (3)	0.032 (2)	0.0030 (19)	0.0055 (17)	0.0057 (19)
C13	0.031 (2)	0.029 (3)	0.026 (2)	−0.0010 (19)	0.0017 (17)	−0.0041 (18)
C14	0.035 (2)	0.024 (3)	0.031 (2)	0.0021 (18)	0.0043 (18)	0.0002 (18)

### Geometric parameters (Å, º)

**Table:**

Cl1—C2	1.709 (5)	C5—H5	0.9500
Cl2—C2	1.718 (5)	C6—C7	1.381 (7)
Cl3—C10	1.733 (5)	C7—C8	1.388 (7)
Cl4—C12	1.735 (5)	C7—H7	0.9500
F1—C6	1.357 (6)	C8—H8	0.9500
N1—N2	1.269 (6)	C9—C10	1.393 (7)
N1—C1	1.416 (6)	C9—C14	1.398 (7)
N2—C9	1.426 (6)	C10—C11	1.403 (7)
C1—C2	1.369 (7)	C11—C12	1.380 (7)
C1—C3	1.490 (6)	C11—H11	0.9500
C3—C8	1.399 (7)	C12—C13	1.399 (7)
C3—C4	1.404 (7)	C13—C14	1.384 (7)
C4—C5	1.390 (7)	C13—H13	0.9500
C4—H4	0.9500	C14—H14	0.9500
C5—C6	1.375 (7)		
			
N2—N1—C1	113.8 (4)	C8—C7—H7	120.7
N1—N2—C9	112.8 (4)	C7—C8—C3	120.8 (5)
C2—C1—N1	112.9 (4)	C7—C8—H8	119.6
C2—C1—C3	123.4 (4)	C3—C8—H8	119.6
N1—C1—C3	123.6 (4)	C10—C9—C14	119.2 (4)
C1—C2—Cl1	123.8 (4)	C10—C9—N2	116.7 (4)
C1—C2—Cl2	122.9 (4)	C14—C9—N2	124.1 (5)
Cl1—C2—Cl2	113.3 (3)	C9—C10—C11	121.0 (4)
C8—C3—C4	118.7 (4)	C9—C10—Cl3	120.9 (4)
C8—C3—C1	121.2 (4)	C11—C10—Cl3	118.1 (4)
C4—C3—C1	120.1 (4)	C12—C11—C10	118.4 (5)
C5—C4—C3	120.8 (4)	C12—C11—H11	120.8
C5—C4—H4	119.6	C10—C11—H11	120.8
C3—C4—H4	119.6	C11—C12—C13	121.8 (5)
C6—C5—C4	118.6 (5)	C11—C12—Cl4	119.3 (4)
C6—C5—H5	120.7	C13—C12—Cl4	118.9 (4)
C4—C5—H5	120.7	C14—C13—C12	118.9 (4)
F1—C6—C5	118.8 (5)	C14—C13—H13	120.5
F1—C6—C7	118.7 (5)	C12—C13—H13	120.5
C5—C6—C7	122.5 (5)	C13—C14—C9	120.7 (5)
C6—C7—C8	118.7 (5)	C13—C14—H14	119.6
C6—C7—H7	120.7	C9—C14—H14	119.6
			
C1—N1—N2—C9	179.1 (4)	C6—C7—C8—C3	−0.8 (7)
N2—N1—C1—C2	−179.2 (4)	C4—C3—C8—C7	0.9 (7)
N2—N1—C1—C3	0.0 (7)	C1—C3—C8—C7	179.1 (4)
N1—C1—C2—Cl1	−178.0 (4)	N1—N2—C9—C10	168.4 (4)
C3—C1—C2—Cl1	2.8 (7)	N1—N2—C9—C14	−13.2 (7)
N1—C1—C2—Cl2	1.7 (6)	C14—C9—C10—C11	0.6 (7)
C3—C1—C2—Cl2	−177.6 (4)	N2—C9—C10—C11	179.1 (4)
C2—C1—C3—C8	50.4 (7)	C14—C9—C10—Cl3	180.0 (4)
N1—C1—C3—C8	−128.7 (5)	N2—C9—C10—Cl3	−1.6 (6)
C2—C1—C3—C4	−131.3 (5)	C9—C10—C11—C12	−0.2 (7)
N1—C1—C3—C4	49.5 (6)	Cl3—C10—C11—C12	−179.6 (4)
C8—C3—C4—C5	−0.9 (7)	C10—C11—C12—C13	−0.1 (7)
C1—C3—C4—C5	−179.2 (4)	C10—C11—C12—Cl4	179.5 (4)
C3—C4—C5—C6	1.0 (7)	C11—C12—C13—C14	0.1 (7)
C4—C5—C6—F1	179.7 (4)	Cl4—C12—C13—C14	−179.6 (4)
C4—C5—C6—C7	−0.9 (7)	C12—C13—C14—C9	0.3 (7)
F1—C6—C7—C8	−179.8 (4)	C10—C9—C14—C13	−0.7 (7)
C5—C6—C7—C8	0.9 (7)	N2—C9—C14—C13	−179.0 (4)

### Hydrogen-bond geometry (Å, º)

*Cg*2 is the centroid of the C9–C14 2,4-dichlorophenyl ring.

**Table:**

*D*—H···*A*	*D*—H	H···*A*	*D*···*A*	*D*—H···*A*
C4—H4···N2^i^	0.95	2.53	3.265 (6)	134
C12—Cl4···*Cg*2^ii^	1.74 (1)	3.92 (1)	3.569 (6)	66 (1)

Symmetry codes: (i) *x*−1, *y*, *z*; (ii) *x*+1, *y*, *z*.
